# Application of ultrasound in periodontics: Part II

**DOI:** 10.4103/0972-124X.44096

**Published:** 2008

**Authors:** Vivek K. Bains, Ranjana Mohan, Rhythm Bains

**Affiliations:** 1 *Senior Lecturer, Department of Periodontics, Saraswati Dental College and Hospital, Lucknow (UP), India*; 2 *Professor and Head, Department of Periodontics, Saraswati Dental College and Hospital, Lucknow (UP), India*; 3 *Senior Lecturer, Department of Conservative Dentistry, Career PG Institute of Dental Sciences and Hospital, Lucknow (UP), India*

**Keywords:** LIPUS, microstreaming, microultrasonics, piezosurgery, ultrasonic cleaner, ultrasound probe

## Abstract

Ultrasound offers great potential in development of a noninvasive periodontal assessment tool that would offer great yield real time information, regarding clinical features such as pocket depth, attachment level, tissue thickness, histological change, calculus, bone morphology, as well as evaluation of tooth structure for fracture cracks. In therapeutics, ultrasonic instrumentation is proven effective and efficient in treating periodontal disease. When used properly, ultrasound-based instrument is kind to the soft tissues, require less healing time, and are less tiring for the operator. Microultrasonic instruments have been developed with the aim of improving root-surface debridement. The dye/paper method of mapping ultrasound fields demonstrated cavitational activity in an ultrasonic cleaning bath. Piezosurgery resulted in more favorable osseous repair and remodeling in comparison with carbide and diamond burs. The effect of ultrasound is not limited to fracture healing, but that bone healing after osteotomy or osteodistraction could be stimulated as well.

## INTRODUCTION

Catuna[1] was first to use ultrasound technology in dentistry in 1953 for cavity preparation. Ultrasound was first introduced in periodontal procedure as ultrasonic scalers in 1955 by Zinner[2] and has undergone many changes since then, and simple compact devices have replaced large, heavy units. The single, bulky universal tip has been replaced by a variety of site specific, slimmer tips (some of which have been coined as microultrasonic).[3]

Ultrasound offers great potential in development of a noninvasive periodontal assessment tool that would offer great yield real time information, regarding clinical features such as pocket depth, attachment level, tissue thickness, histological change, calculus, bone morphology, as well as evaluation of tooth structure for fracture cracks.[4]

Spranger[5] was first to try ultrasonography in periodontology, he tried to determine height of alveolar crest in periodontal patients. Palou *et al.*[6,7] attempted to image the crest of alveolar bone by aiming ultrasound transducer perpendicular to long axis of tooth. Eger *et al.*[8] assessed the measurement of gingival thickness using an ultrasonic device with a 5 MHz transducer. While these efforts proved the feasibility of ultrasonic imaging in dentistry, investigators showed that ultrasonic device measures echoes from the hard tissue of tooth surface, and periodontal attachment level can be inferred from these echoes.[9,10] Periodontal structures mapping system using ultrasonic probe was invented at NASA by Companion under supervision of Joseph Heyman and patented in May 1998.[11] Reports also suggest role of ultrasound in osteopromotion[12] and in piezoelectric bone surgery.[13] This paper is intended to review the various applications of ultrasound in periodontics.

## APPLICATION OF ULTRASOUND IN DIAGNOSIS

### Periodontal ultrasonography

Roots for the ultrasound technology used in periodontal ultrasonography are in an ultrasound-based time-of-flight technique used routinely in NASA Langley Research Center's Non-Destructive Evaluation Sciences Laboratory to measure material thickness and, in some cases, length.[3] The primary applications of that technology have been in aircraft skin thickness for corrosion detection and bolt length for bolt tension measurements.[3] Ultrasound probe works somewhat like a sonogram. With a sonogram, the probe is pressed against the body and the beam penetrates the womb and the echoes that come back are recorded and displayed as an image of the fetal face. Same technology is adapted to image the periodontal structures, mainly by making the probe that sends the ultrasound signals and receives the echoes very small. Software in computer sorts out all of echoes and makes an image of attachment level pocket depth, etc. automatically.[14]

Current method of diagnosing periodontal disease (walking probe) is invasive, uncomfortable, and inexact. Ultrasonography probe provides a mapping system for noninvasively making and recording differential measurements of depth of any patient's periodontal ligaments relative to a fixed point as cementoenamel junction. Mapping system uses ultrasound to detect top of ligaments at various points around each tooth and uses either ultrasound or an optical method to find CEJ at same points. Depth of sulcus is calculated as difference between the points.[3]

### Periodontal ultrasonic probe

It consists of transducer, which is housed within a contra angled handpiece at the base of hollow conical tip. This is responsible for emitting and receiving sound waves. Hollow tip focuses acoustic beam into periodontal tissue. Transducer is mounted at the base of a dual taper, convergent–divergent coupler in order to provide acoustically tapered interface with a throat area in order of 0.5 mm. A throat area of 1.5 mm represents an active area reduction from transducer element to aperture. Such a reduction is mandated by geometry and very small window offered by gingival margin. An added virtue of attaining a small tip size is ability of ultrasonic probe to examine interdental area.[4,9,14,15]

Equipments required to run the probe include computer, monitor, keyboard, separate electron box for water pressure control, and foot pedal – all mounted on large cart to transport conveniently. Probe tip incorporates a slight flow of water to ensure a good coupling of ultrasonic energy to tissues. Water can come either from a suspended IV-type sterile bag or plumbed from dental chair.[7]

### Working of ultrasonic probe

Ultrasonic probe tip is held in a vertical position, parallel to long axis of the tooth. Tip is gently placed on gingival margin until slight blanching of gingiva is visualized; ensuring the complete coupling of water into gingival sulcus, the probe is activated with a foot pedal. When foot pedals are engaged, a small stream of water will flow into sulcus along with a thin beam of ultrasound waves. Ultrasonic probe projects a narrow frequency (1–20 MHz) ultrasonic pulse into gingival sulcus or periodontal pocket and then detects echoes of returning wave. An ultrasonic beam entering the tissues is absorbed, reflected, or scattered. Reflected portion is received by machine and used for reconstruction of ultrasonic image. As these sound waves bounces of periodontal tissues, echoes are recorded by a tiny transducer in handpiece and analyzed simultaneously by computer attached to ultrasonic unit. As the examiner passes probe tip across the gingival margin, computer records incoming data which employs artificial intelligence algorithms to translate out data into estimates of probing depth in millimeters.[4,14]

Unlike manual probing where measurements are obtained at six sites per tooth, ultrasound probe tip is gently placed on gingival margin then swept along entire gingival area. Thus, the probe is able to painlessly capture a series of observations (depth measurements and contour) across entire subgingival area as probe tip passes gingival margin so yielding more information.[4] Though this noninvasive method for measuring pocket seems to be accurate long-term evidence-based studies are needed.

## APPLICATION OF ULTRASOUND IMAGING FOR PERIODONTAL ASSESSMENT

A recent study using the ULTRADERM^®^ (Longport International Ltd, Silchester, U.K.) ultrasonic scanner that works at the frequency of 20 MHz in an animal (pig jaw) model had shown that periodontal ultrasonography can produce images suitable for the assessment of the periodontium as well as accurate measurement of the dimensional relationship between hard and soft structures.[16,17] The technique of ultrasonography has also been applied in human subjects. In various studies the device was used to evaluate gingival thickness before and after mucogingival therapy for root coverage.[17,18] It was also used to assess the dynamics of mucosal dimensions after root coverage with connective tissue grafts,[17,19] bioresorbable barrier membranes,[17,20] and for measurement of masticatory mucosa.[17,21] Ultrasonic scanner provides satisfactory results both in terms of accuracy and repeatability.[16]

## APPLICATION OF ULTRASOUND IN CALCULUS DETECTION

There are a variety of subgingival calculus detection systems in the market as Detectar^®^, Keylaser II^®^, and Dental Endoscope[22] and for *in vitro* studies.[23,24] Meissner *et al.*[25] developed an ultrasound-based device for office use which was automatically able to detect subgingival calculus. They showed that dental surfaces may be discriminated by the analysis of tip oscillations of an ultrasonic instrument, which possesses computerized calculus detection (CCD) features. This system may help to judge other systems *in vivo*.

## APPLICATION OF ULTRASOUND IN SCALING AND ROOT PLANING

### The mechanism of removing calculus

Cavitation was assumed to be able to liberate some energy for the removal of deposits. However, it has been found that using only the cavitation without the touch of the vibrating tip is not sufficient to remove the calculus, and that a direct contact between the vibrating tip and the calculus is necessary.[26] Therefore, it is now generally accepted that the mechanical energy produced by the vibrating tip (chipping action) along with cavitation is responsible for the removal of deposits.[27–29]

### Mechanism of removing biofilm or plaque

Cavitational activity has an adverse effect on bacteria within dental plaque.[30] The effect of this cavitational activity disrupts bacterial cell wall and subgingival microbial environment.[31] Microstreaming or acoustic mainstreaming, generated by ultrasound in the presence of a fluid environment, also is effective in removing bacterial plaque.[3,32] Ultreo^®^ (Inc. today) is a revolutionary power toothbrush that combines ultrasound waveguide technology with precisely tuned sonic bristle action for a deep, gentle, long-lasting feeling of clean. Clinical studies prove that Ultreo can remove up to 95% of plaque from hard-to-reach areas in the first minute of brushing.[33]

### Endotoxin removal and root detoxification

Currently, it is understood that endotoxin (LPS) is a surface substance which is superficially associated with the cementum and calculus and that it is easily removed by washing, brushing, light scaling, or polishing the contaminated root surface.[32,34–39] Heat automatically generated from magnetostrictive units may assist in endotoxin removal or detoxification, as a result, areas of the tooth where the tip does not touch may inadvertently be detoxified as well.[3]

### Cautions while using ultrasonics in scaling and root planing

As with any dental procedures universal precautions consists of wearing protective eyewear and/of face shield, mask and gloves. In the beginning of the day, hold the handpiece over a sink or drain, set power to low, activate the foot control, and flush the water line of unit at maximum water-flow for at least 2 minutes to clear stagnant water and reduce water films in tubing.[3] All the inserts are autoclavable and should be sterilized before every use. Having the patient rinse with approved antimicrobials, and high vacuum suction, helps to minimize aerosols. An aerosol reduction device (ARD) that is attached to the ultrasonic handpiece has been shown to reduce contamination cloud by placing suction in close proximity to the ultrasonic tip.[40] Harrel *et al.*[41] showed a high volume evacuator attachment to an ultrasonic handpiece significantly reduced the detectable aerosols splatter produced during ultrasonic scaling by 93%. Veksler *et al.*[42] reported 30-second rinse with an essential oil mouthrinse before instrumentation reduces bacterial count in the aerosol by 92.1% and salivary bacterial level by about 50% for up to 40 minutes and 97% reduction for up to 60 minutes following two 30-second rinses with 0.12% chlorhexidine. Aerosols can be suspended in air for up to 30 minutes after using power-driven scalers,[43] so, infection control is to be maintained even after the procedure is finished. A light pen grasp, and fulcrum (intra or extraoral) should be used. Lightest amount of lateral pressure possible while still maintaining control of instrument and proper adaptation of tip is necessary for calculus removal.[3] Increasing the pressure decreases the mechanical vibrations, the chipping action, and ultimately the effectiveness of ultrasonics.[44] To prevent root damage in SRP the assessed magnetostrictive ultrasonic scaler should be the used at 0.5 N lateral pressure, low or medium power setting, and tip close to zero degree angulation with tooth.[44] Increasing the power also increases aerosol formation, which results in reduced water cooling and cavitational effect and can increase patient sensitivity. Chapple *et al.*[45] showed that use of half power setting was as effective as using the ultrasonic scaler at full power. Ultrasonic tips are now shaped more like probes. Insert the tip vertically, parallel to long axis of tooth, walk the tip into proximal surfaces, keeping the tip adapted to tooth-like probe. To ensure patient comfort, keep the tip moving at all the times when in contact with tooth.[3] Direct vision is preferred when using ultrasonics, as indirect vision is compromised by mist of irrigant. Ultrasonic tips do not last forever. As tip wear, scaling efficiency decreases. One millimeter of tip wear results in approximately 25% loss of efficiency and 2 mm of wear results in approximately 50% loss of efficiency and at this point tip should be replaced.[3,46] Clinicians who retain their inserts for years may find their instrument tips become reduced in length through wear, potentially leading to a detrimental change in performance of scaling system.[47] The instrument should be kept away from bone to avoid possibility of necrosis and sequestration.[48]

## APPLICATION OF ULTRASOUND IN CURETTAGE

Ultrasound is effective for debriding the epithelial lining of periodontal pockets resulting in microcauterization. Morse scaler-shaped and rod-shaped ultrasonic instruments are used for this purpose.[49] Investigators found ultrasonic instruments to be as effective as manual curettage[50–52] and less inflammation and less removal of connective tissue.[49]

## APPLICATION OF ULTRASOUND ASMICROULTRSONICS

Kwan[53] and Peggy Hawkins[54] coined the term ‘microultrasonics’ in early 1990s as a generic term that identifies the refined use of powered instrumentation in supragingival and gross debridement. Microultrasonics is innovative, power-driven (magnetostrictive or piezoelectric) scaler products featuring thinner tips and varied shapes. These are small, approximating the size of a periodontal probe and can be used for supra and subgingival treatment at low to high power, with less to no water spray, and little or no adjunctive use of hand instrumentation.[3,53,54]

Tips of microultransonic instruments measure about 0.2–0.6 mm in diameter. These are powered to move up to ultrasonic speeds (25000 to more than 40000 cycles per second), with active working sides on all surfaces of vibrating instrument and provide ultrasonically activated lavage in working field.[53] Ultrasonic motors need no deceleration mechanism because they have high torque output in low rotation speed.[55] Microultrasonic instruments are less likely to overinstrument roots because they are neither sharp nor abrasive.[53]

The periodontal endoscope allows for visual access to root surfaces with great magnification, lessening the need for surgical intervention. Combined with a simple array of microultrasonic instruments, endoscopic debridement can be accompanied in a nonsurgical, minimally invasive way by the dentist or periodontist.[53]

## APPLICATION OF ULTRASOUND AS ULTRASONIC CLEANER

The dye/paper method of mapping ultrasound fields demonstrated cavitational activity in an ultrasonic cleaning bath.[56] The ultrasonic cavitation implosion effect is incredibly effective in displacing the saturated layer of contaminant, allowing fresh cleaning solvent to come in contact with the unsaturated surface to attack and dissolve the remaining contaminant. It is especially effective on unsmooth and out-of-reach surfaces that are normally inaccessible through conventional means such as brushing. It has been shown to speed and enhance the effect of numerous chemical reactions. The most probable reason for this enhancement is due to the levels of high energy created by the high temperatures and pressure emitted by millions of individual cavitation bubble implosions.[57] In view of the use of such baths as a part of the process of controlling cross-infection in dentistry, it is suggested that manual cleaning of reusable instruments may be necessary as well.[56]

**Figure 1:**
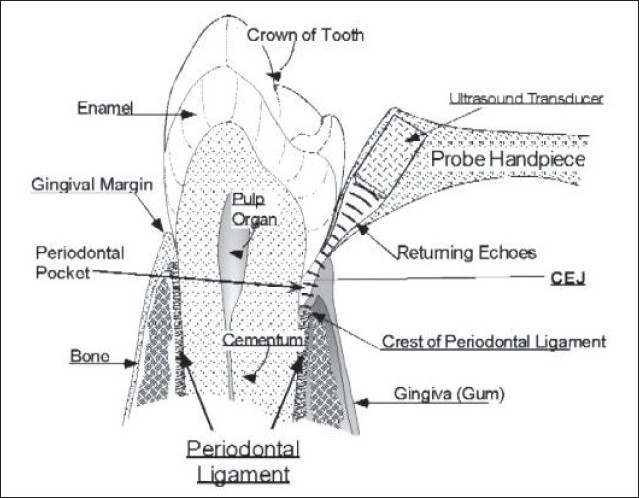
Ultrasonic periodontal probe with probe tip at the gingival margin and ultrasound projected into the periodontal pocket. The echoes returning from the crest of the periodontal ligament are shown (Hinders M And Companion J; www.acoustics.org)

**Figure 2:**
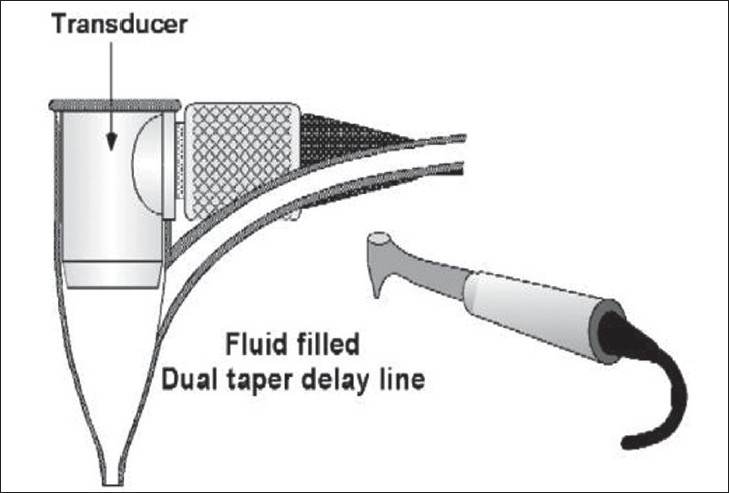
The hand piece contains a probe tip, which is small enough to permit scanning of the area between teeth. The ultrasonic transducer is mounted in a probe tip shell (a fluid-filled dual taper delay line), which has a throat diameter small enough to project the ultrasonic beam into the narrow space between the tooth and bone (Hinders M And Companion J; www.acoustics.org)

**Figure 3:**
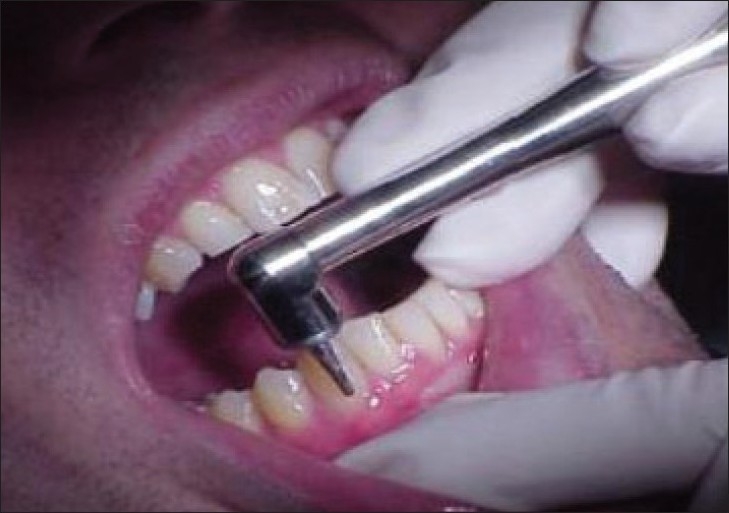
Ultrasonographic periodontal probe in use; (as.wm.edu/Faculty/Hinders/NDE-Projects.html)

**Figure 4:**
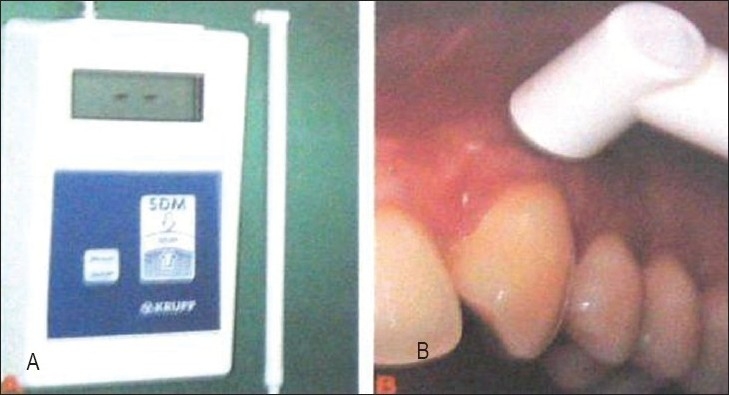
Ultrasonic device (B) measuring gingival thickness at a grafted recession site. (Muller H P, Stahl M, Eger T; 1999)

**Figure 5:**
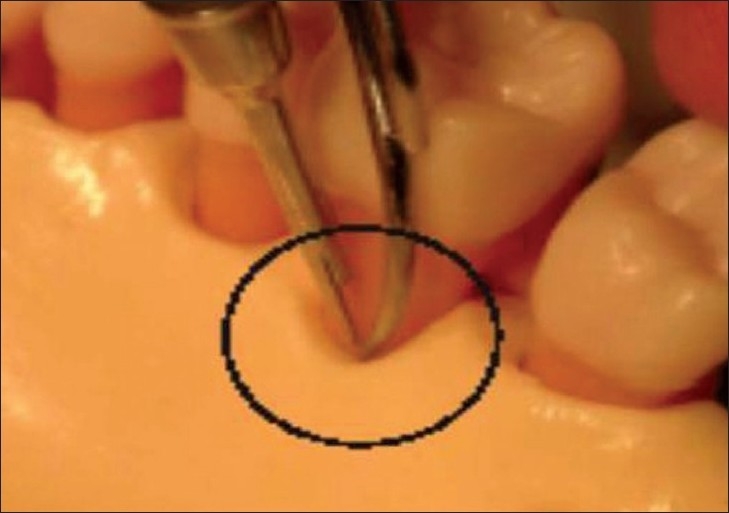
Camera and micro ultrasonics for endoscopic instrumentation; (Kwan J Y; 2005)

**Figure 6:**
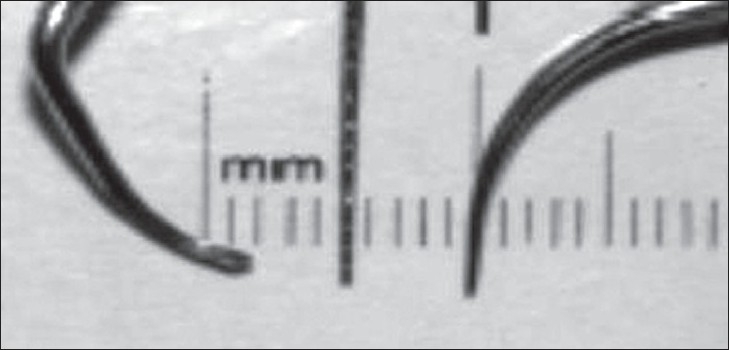
Scaler, probe and microultrasonic insert. (Kwan J Y; 2005)

## APPLICATION OF ULTRASOUND IN BONE SURGERY

Ultrasonic bone-cutting surgery has been recently introduced as a feasible alternative to conventional tools of craniomaxillofacial surgery, due to its technical characteristics of precision and safety.[58] Piezosurgery^®^ (Mectron Medical Technology, Carasco, Italy) is a new and innovative method that uses piezoelectric ultrasonic vibrations to perform precise and safe osteotomies.[59] It was first invented by Tomaso Vercelotti to overcome the limitations of traditional instruments in oral bone surgery.[60] It was first reported for preprosthetic surgery, alveolar crest expansion, and sinus grafting.[60,61] The equipment consists of a piezoelectric handpiece, and a foot switch that is connected to a main unit, which supplies power, and has holders for handpiece and irrigation fluids. It contains a peristaltic pump for cooling with a jet of solution that discharges from the inserts with an adequate flow of 0–60 ml/minute and removes detritus from the cutting area.[62] Piezoelectric surgery uses a specifically engineered surgical instrument characterized by a surgical power that is 3-times higher than normal ultrasonic instruments.[63] The device used is unique in that the cutting action occurs when the tool is employed on mineralized tissues, but stops on soft tissues.[61] Nerves, vessels, and soft tissues are not injured by the microvibrations (60–200 mm/sec), which are adjusted to target only mineralized tissue.[64] It has variable modulations of frequency (25–30 kHz) that gives inserts a specific vibration that allows the cut to keep clean of bone splinters. The elevation of membrane from the sinus floor is performed using both piezoelectric elevators and the force of a physiologic solution subjected to piezoelectric cavitation.[63] Piezosurgery resulted in more favorable osseous repair and remodeling in comparison with carbide and diamond burs. Also, force necessary to obtain a cut by the operator is much less compared to a rotational bur. Patients perceive greater comfort with this instrument in osseous surgery as it eliminates noise of high-speed handpiece.[65]

**Figure 7:**
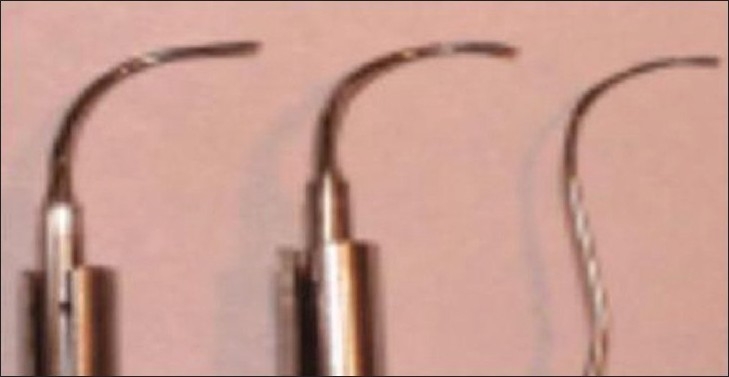
Angled insert, modified curved/ angled insert, and furcation probe. (Kwan J Y; 2005)

## APPLICATION OF ULTRASOUND IN OSTEOCONDUCTION

Since the first therapeutic ultrasound application in 1932, ultrasound therapy has evolved within the physiotherapy practice mainly to treat soft tissue disorders. The first prospective randomized double blind clinical trials were published in the nineties. The results of these studies indicated that the healing time of fresh tibial and radial fractures could be reduced by 38% after ultrasound was applied.[12] Here, low intensity pulsed ultrasound was used with an intensity of 30 mW/cm^2^, 20 minutes a day, by placing a transducer onto the skin across the fracture. It also became clear that slow or nonuniting fractures could be healed by the application of ultrasound. Furthermore, it seemed that the effect of ultrasound is not limited to fracture healing, but that bone healing after osteotomy or osteodistraction could be stimulated as well. It was, therefore, concluded that there may be a potential for ultrasound to stimulate maxillofacial bone healing.[12] Based on the literature, it seems to be reasonable to assume that ultrasound has an effect on bone cells during bone healing, but that a possible observed effect may be related to the mechanical and circulatory conditions at the site. However, Schortinghuis J *et al*,[66] showed that low intensity pulsed ultrasound is not effective in stimulating bone growth into a rat mandibular defect, either with or without the use of osteoconductive membranes. Also, no evidence suggests that low-intensity pulsed ultrasound (LIPUS) stimulates osteoconduction in a bone defect in the rat mandible that is covered by a collagen membrane.[66,67]

## ULTRASOUND MAY HELP TO REGROW TEETH?

High-intensity focused ultrasound has been shown to stop bleeding in blood vessels noninvasively,[68] whereas LIPUS) has been reported to be effective in liberating preformed fibroblast growth factors from a macrophage-like cell line, and it stimulates angiogenesis during wound healing,[69] enhances mandibular growth in growing baboons,[70] enhances bone growth into titanium porous implants,[71] accelerates healing of resorption by reparative cementum,[72] and enhances bone healing after fractures[73] and mandibular osteodistraction.[74] Routine use of low-intensity ultrasound appears to have a modest beneficial effect on recurrent aphthous stomatitis.[75] Reports also suggest that LIPUS can enhance the growth of mandibular incisor apices and accelerate the rate of eruption in teeth that received ultrasound in rabbits.[76] Using LIPUS, Dr Tarak El-Bialy from the faculty of medicine and dentistry and Dr Jie Chen and Ying Tsui from the faculty of engineering have created a miniaturized system-on-a-chip that offers a noninvasive and novel way to stimulate jaw growth and dental tissue healing.[77–79] The prototype ultrasound device can be mounted on braces or a plastic removable crown.[79] The wireless device, roughly half the size of a nail on the baby finger, will be able to gently massage the gums to stimulate growth of the tooth root.[80] LIPUS seems to play an effective role in repair and healing, still their role in periodontal regeneration needs to be investigated. Till now, there are not enough studies that specify the role of LIPUS in periodontal regeneration.

## CONCLUSION

Diagnostic application of mega Hertz ultrasound includes use of ultrasonography for dimensional assessment of periodontal structures. The advantage of using noninvasive assessments without ionizing radiations could lead to applications in the evaluation of soft and hard tissue healing after periodontal surgery (regenerative, mucogingival, or implant surgery) as well as for clinical assessment and treatment planning prior to implant placement. In therapeutics, ultrasonic instrumentation is proven effective and efficient in treating periodontal disease. When used properly, ultrasound is kind to the soft tissues, requires less healing time, and is less tiring for the operator. LIPUS seems to play an effective role in repair and healing, still their role in periodontal regeneration needs to be investigated. Though ultrasound application in both diagnosis and periodontal therapy seems to present promising results, long-term evidence-based studies are required to use ultrasound in routine periodontal practice.
